# Supplementary figures and images for: Scn1a-GFP transgenic mouse revealed Nav1.1 expression in neocortical pyramidal tract projection neurons
Source: eLife. 2023 May 23;12:e87495. doi: 10.7554/eLife.87495 (PMC10205085; doi:10.7554/eLife.87495)

Figure 1B-source data1-1

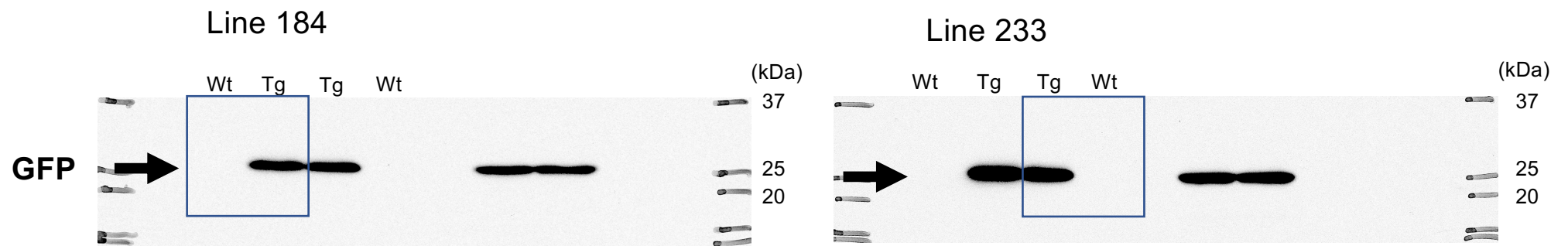

Figure 1B-source data1-2

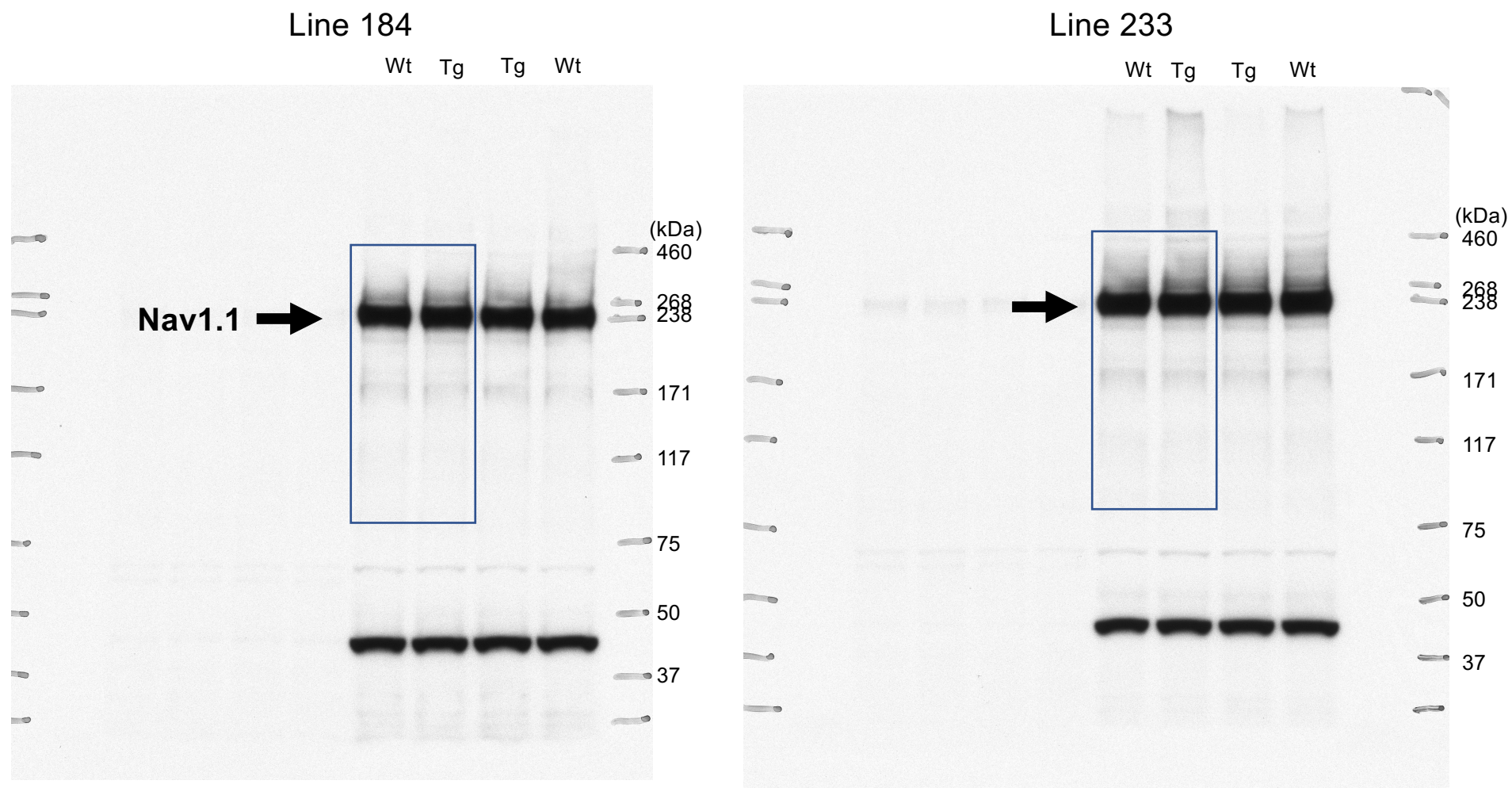

Figure 1B-source data1-3

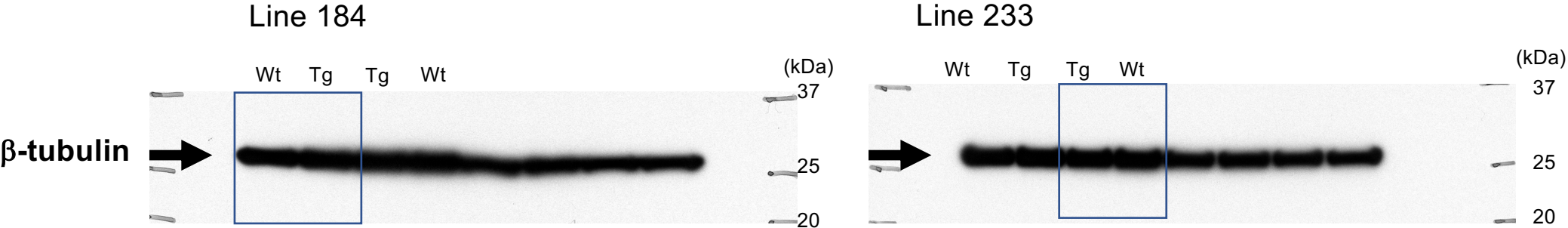

Supplement: Figure 1—source data 1. [file elife-87495-fig1-data1.zip › Figure1-sourse data_1/figure1_Source_data1.pdf]

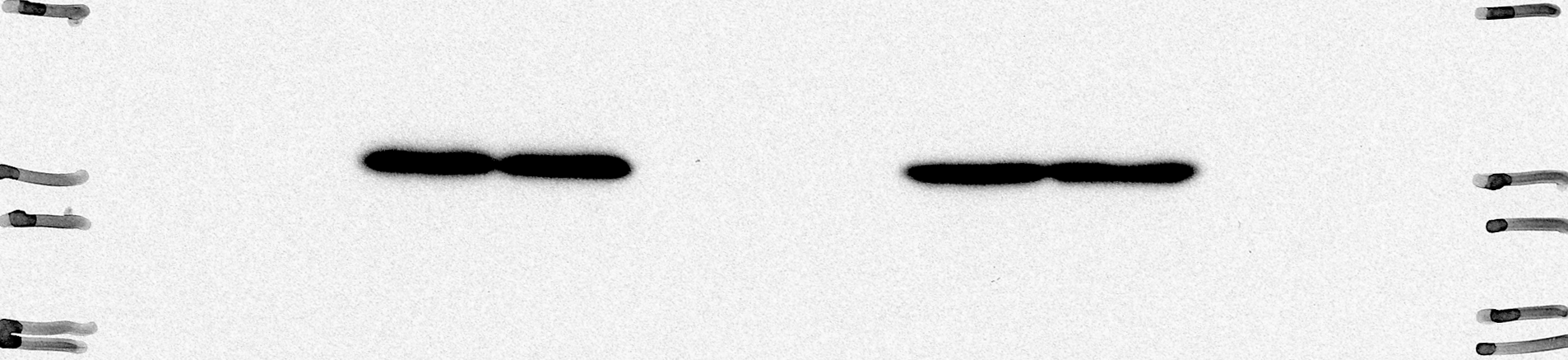

Supplement: Figure 1—source data 1. [file elife-87495-fig1-data1.zip › Figure1-sourse data_1/Uncropped_original_files/1aGFP_184_GFP.tif]

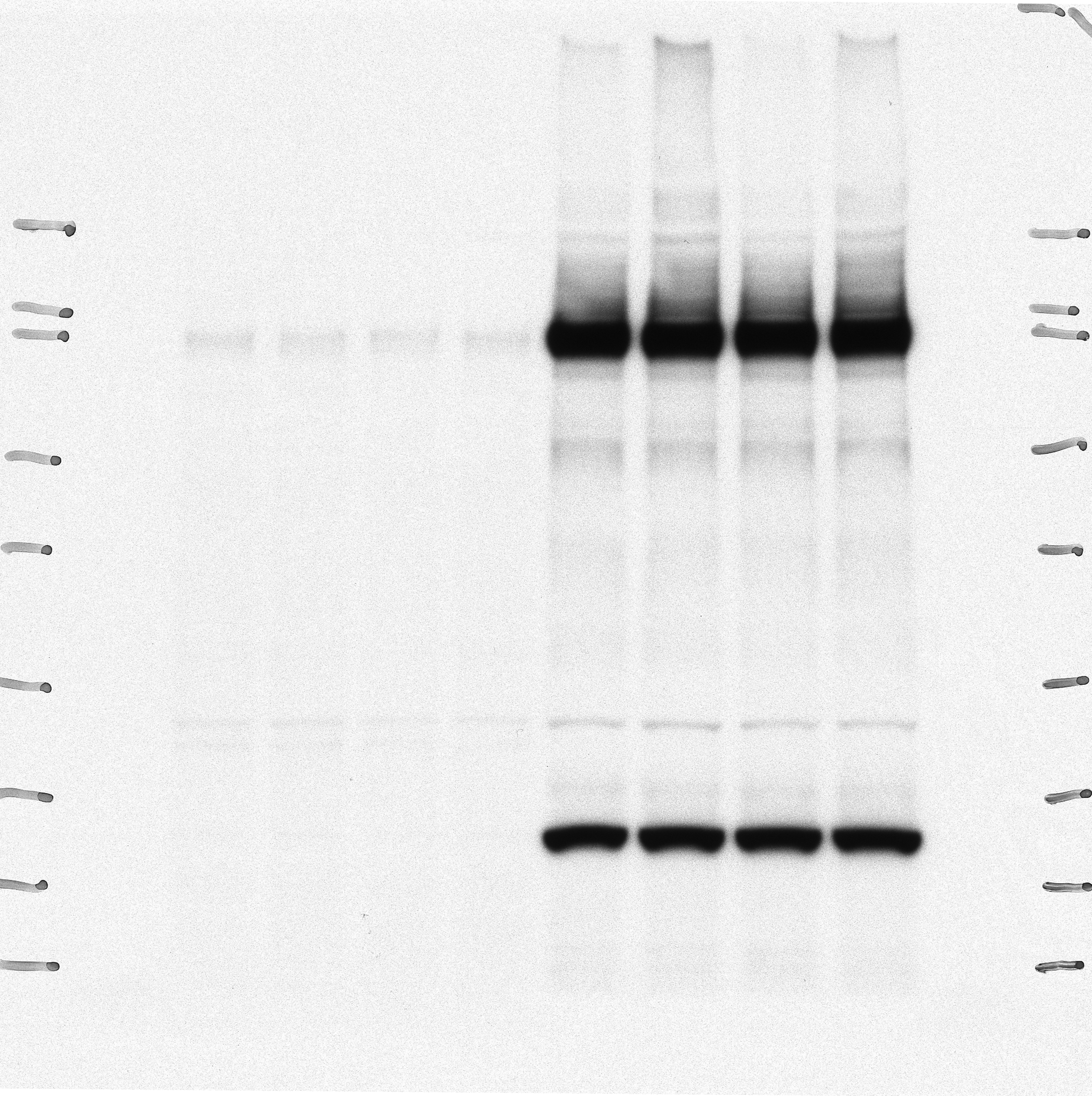

Supplement: Figure 1—source data 1. [file elife-87495-fig1-data1.zip › Figure1-sourse data_1/Uncropped_original_files/1aGFP_184_Nav1.tif]

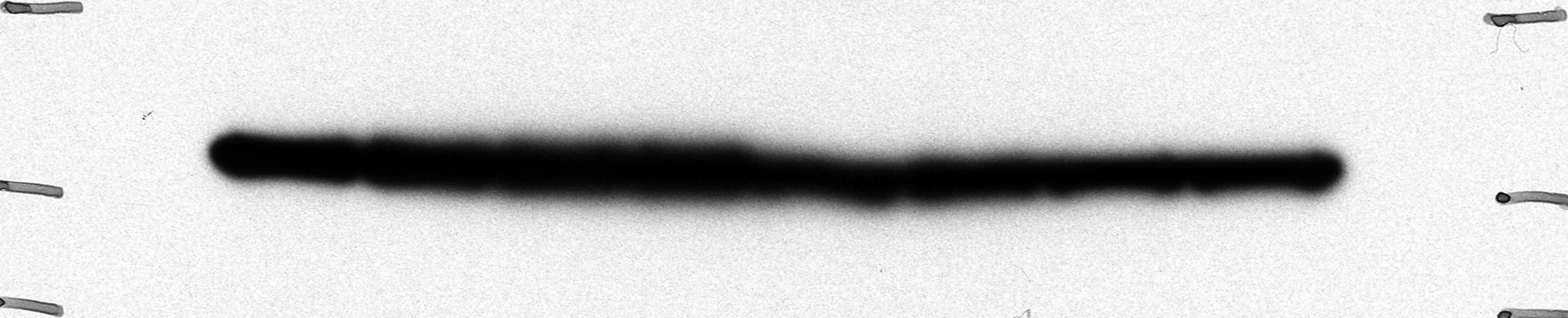

Supplement: Figure 1—source data 1. [file elife-87495-fig1-data1.zip › Figure1-sourse data_1/Uncropped_original_files/1aGFP_184_tubb_2.tif]

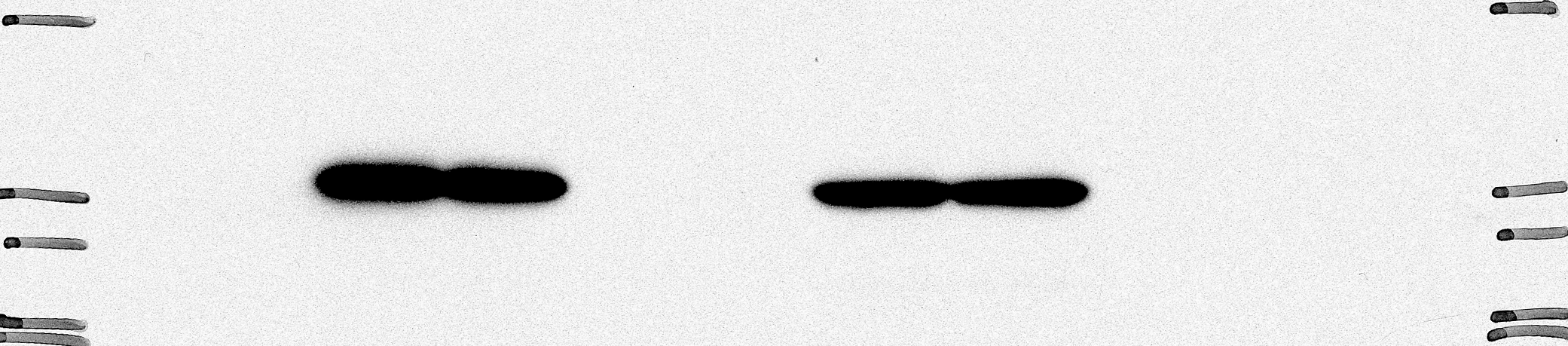

Supplement: Figure 1—source data 1. [file elife-87495-fig1-data1.zip › Figure1-sourse data_1/Uncropped_original_files/1aGFP_233_GFP.tif]

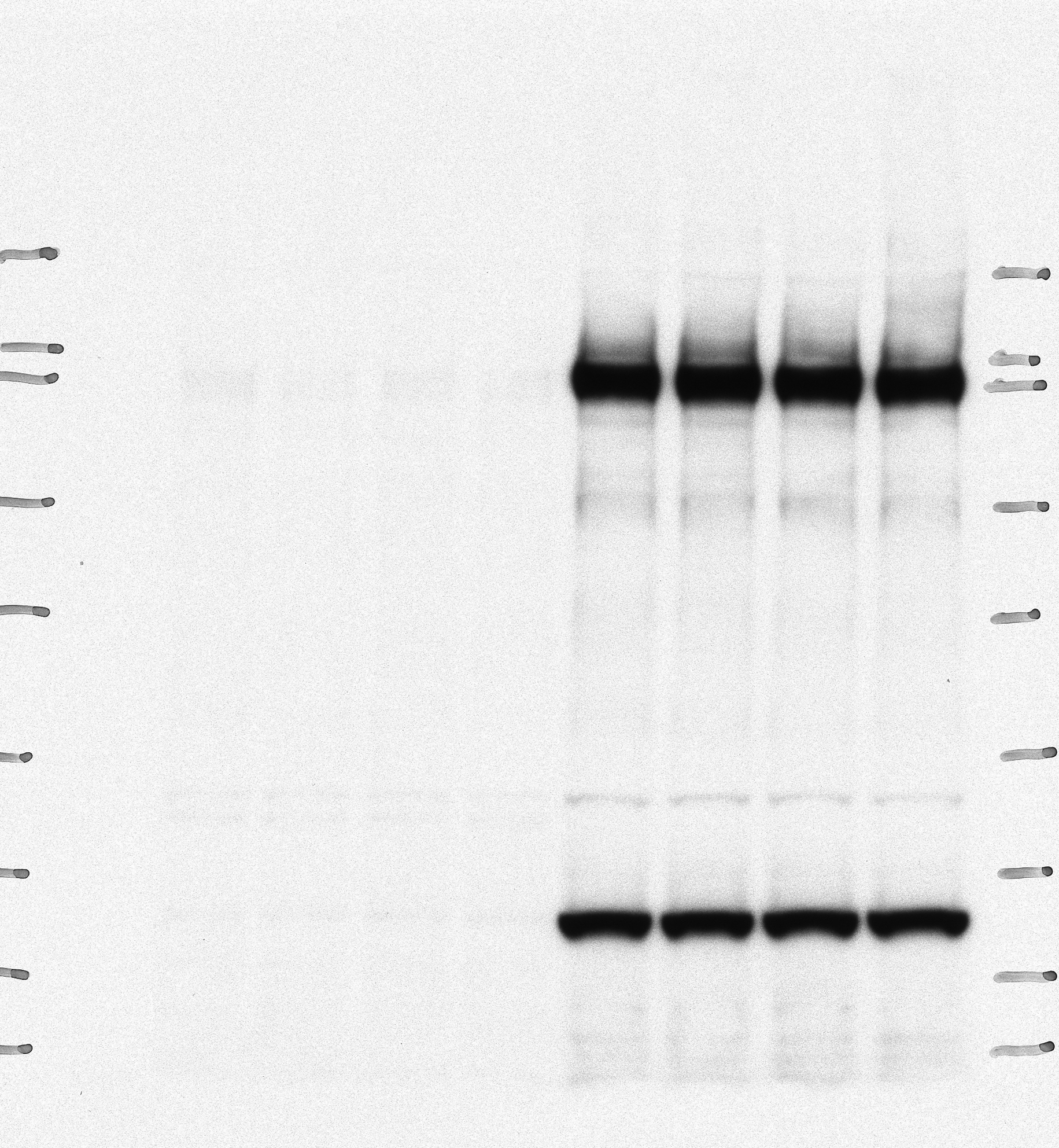

Supplement: Figure 1—source data 1. [file elife-87495-fig1-data1.zip › Figure1-sourse data_1/Uncropped_original_files/1aGFP_233_Nav1.tif]

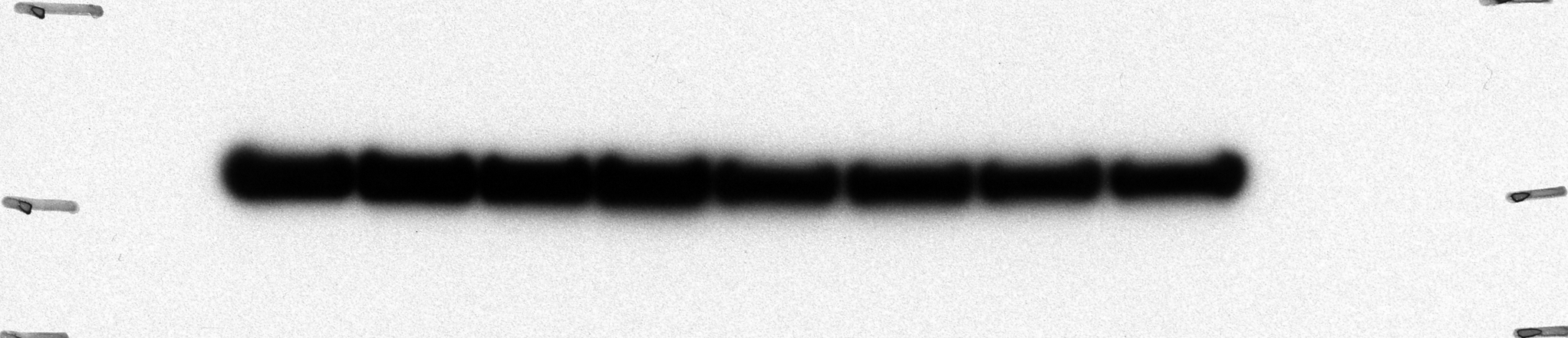

Supplement: Figure 1—source data 1. [file elife-87495-fig1-data1.zip › Figure1-sourse data_1/Uncropped_original_files/1aGFP_233_tubb.tif]

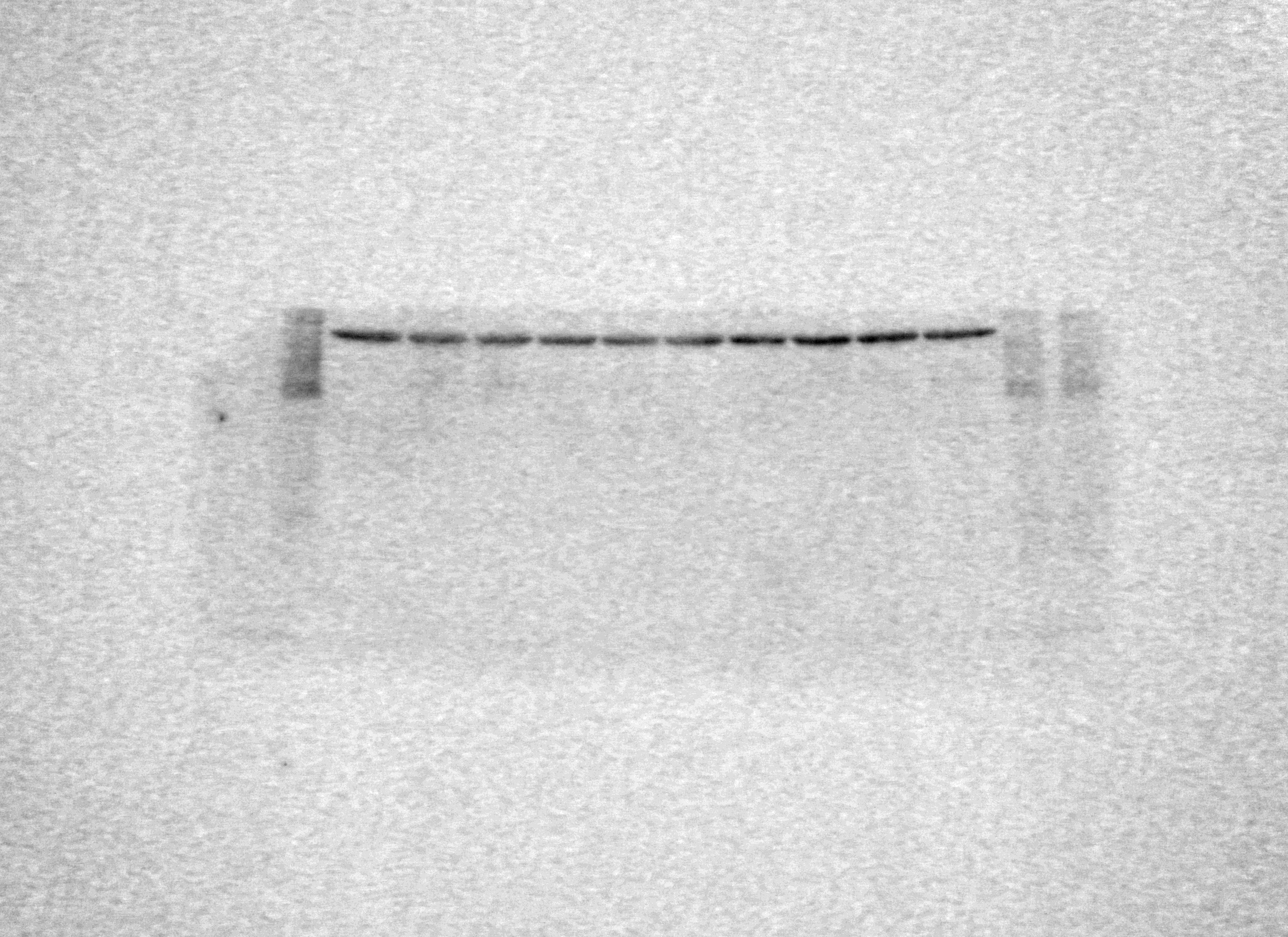

Supplement: Figure 2—figure supplement 2—source data 1. [file elife-87495-fig2-figsupp2-data1.zip › Figure2-figure_supplement_2-source data_1/Uncropped_original_files/GAPDH.tif]

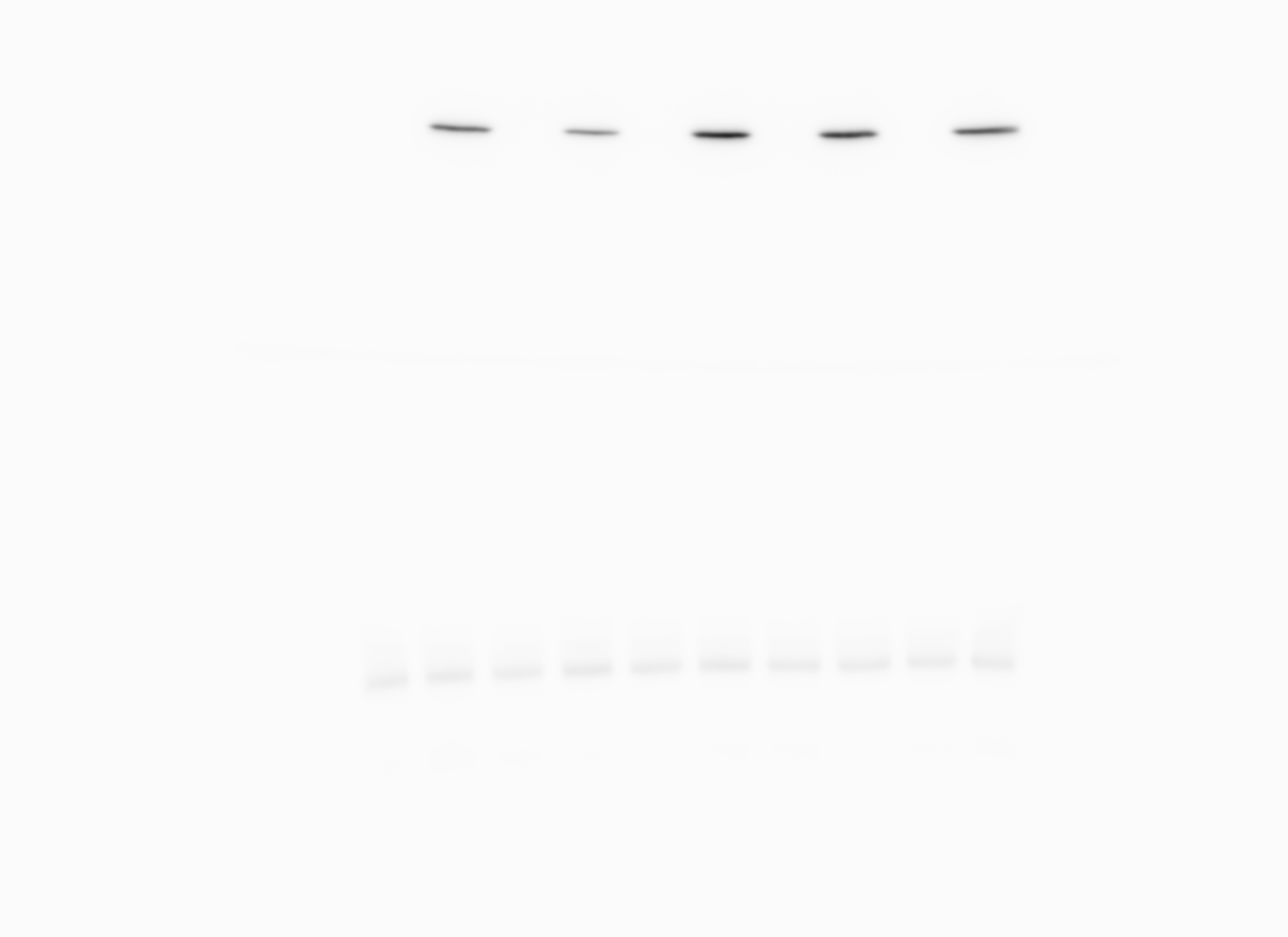

Supplement: Figure 2—figure supplement 2—source data 1. [file elife-87495-fig2-figsupp2-data1.zip › Figure2-figure_supplement_2-source data_1/Uncropped_original_files/GFP.tif]

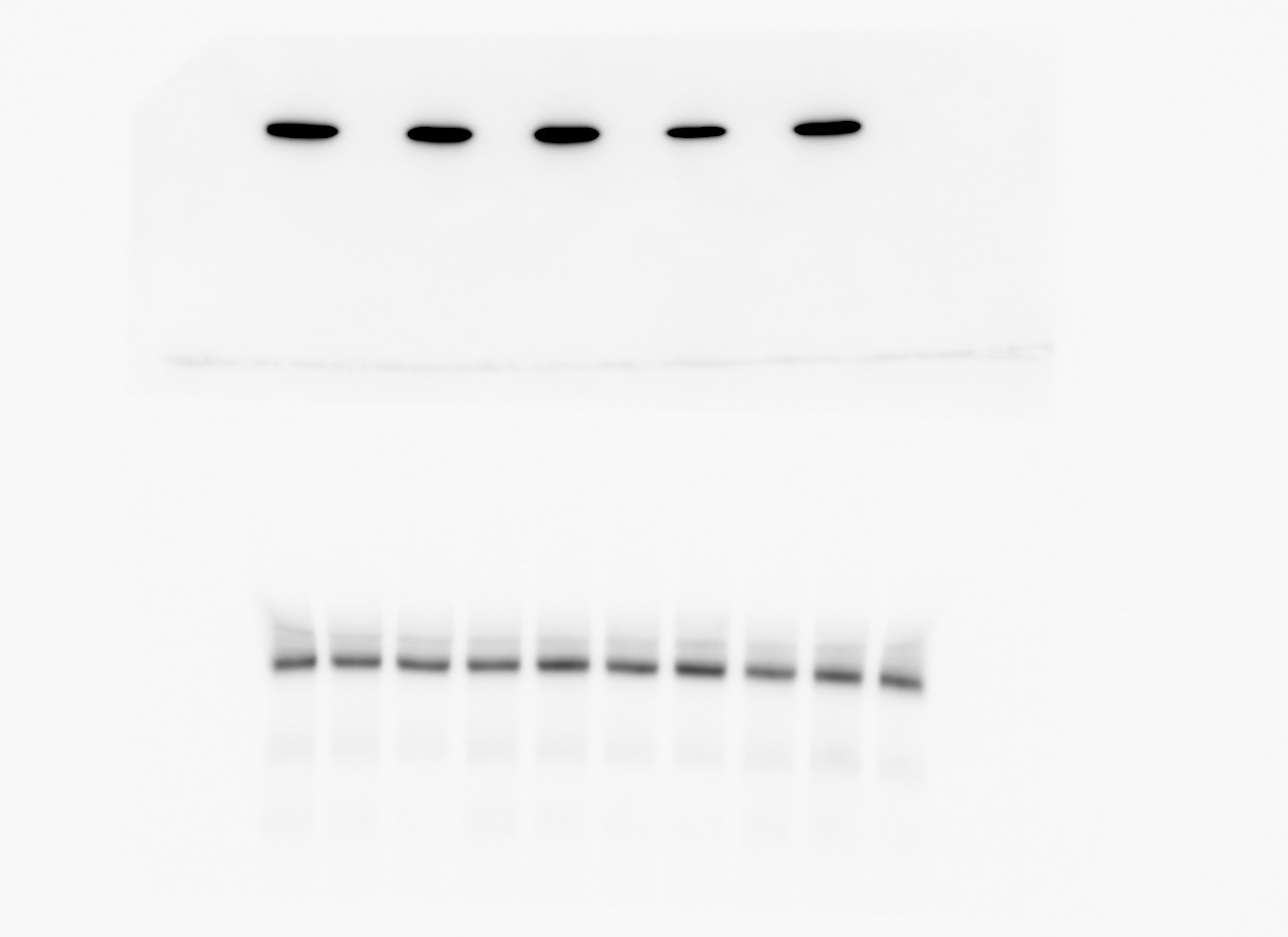

Supplement: Figure 2—figure supplement 2—source data 1. [file elife-87495-fig2-figsupp2-data1.zip › Figure2-figure_supplement_2-source data_1/Uncropped_original_files/Nav1_1.tif]

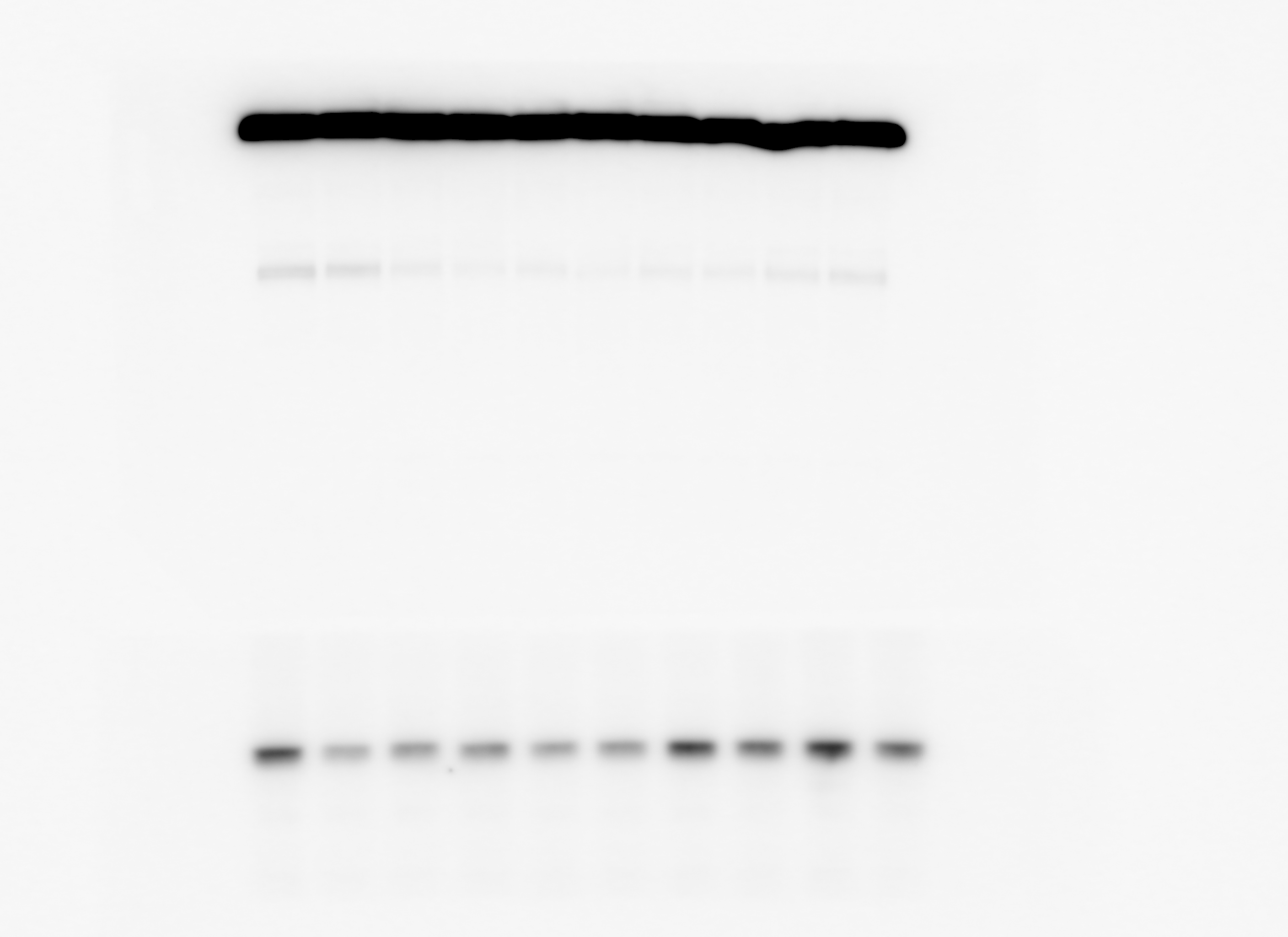

Supplement: Figure 2—figure supplement 2—source data 1. [file elife-87495-fig2-figsupp2-data1.zip › Figure2-figure_supplement_2-source data_1/Uncropped_original_files/tubb.tif]
