# Supplementary material for: Scn1a-GFP transgenic mouse revealed Nav1.1 expression in neocortical pyramidal tract projection neurons
Source: eLife. 2023 May 23;12:e87495. doi: 10.7554/eLife.87495 (PMC10205085; doi:10.7554/eLife.87495)

Figure 2-figure supplement 2-source data1-1

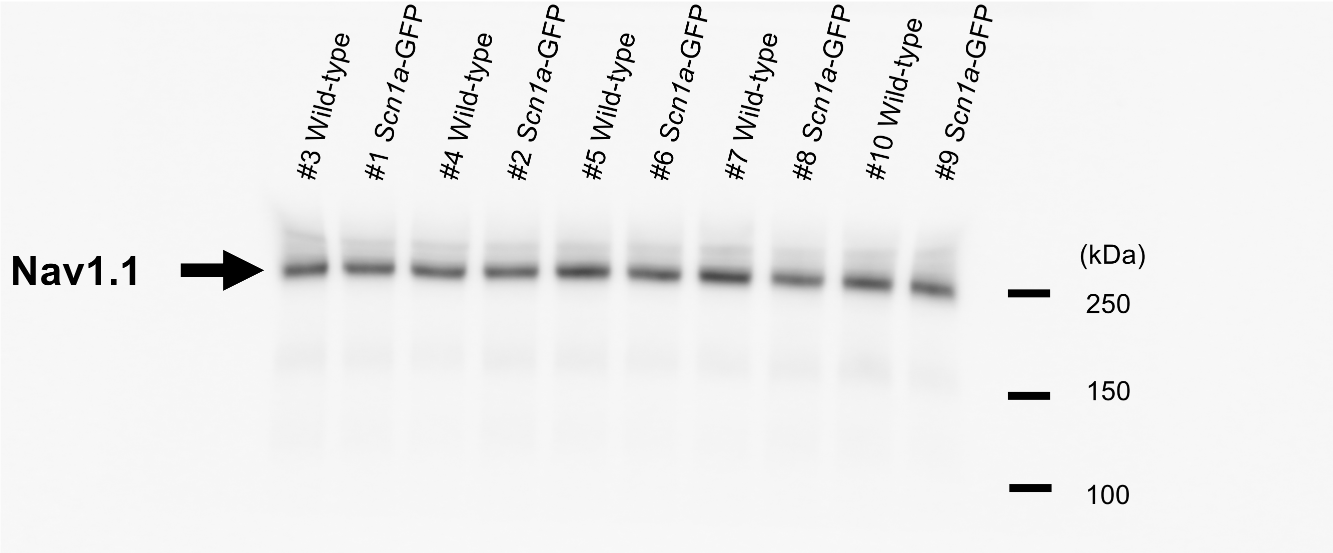

Figure 2-figure supplement 2-source data1-2

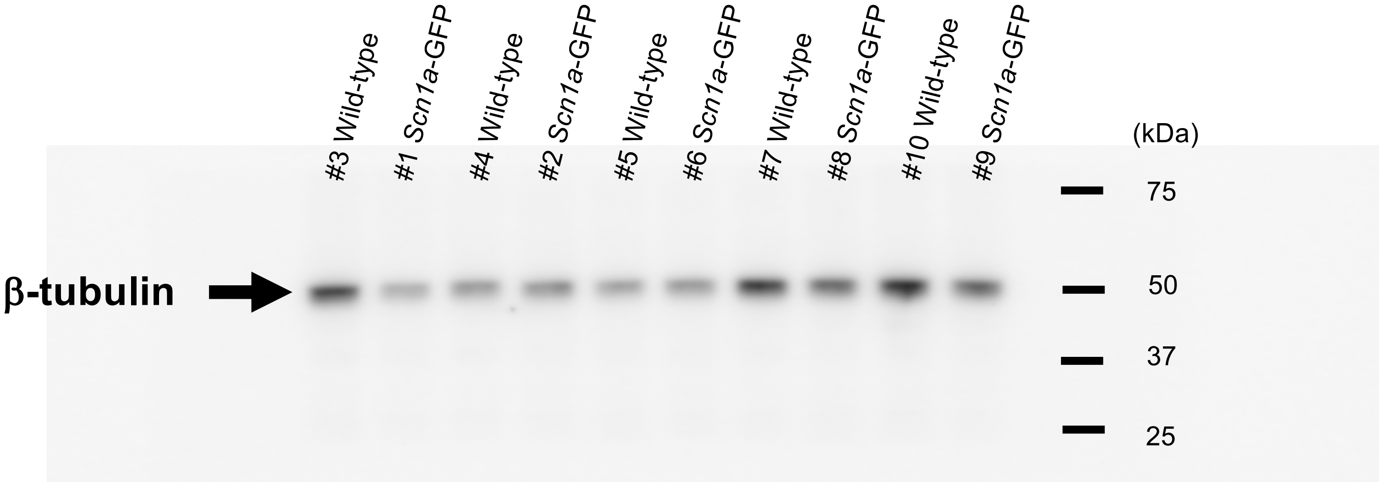

Figure 2-figure supplement 2-source data1-3

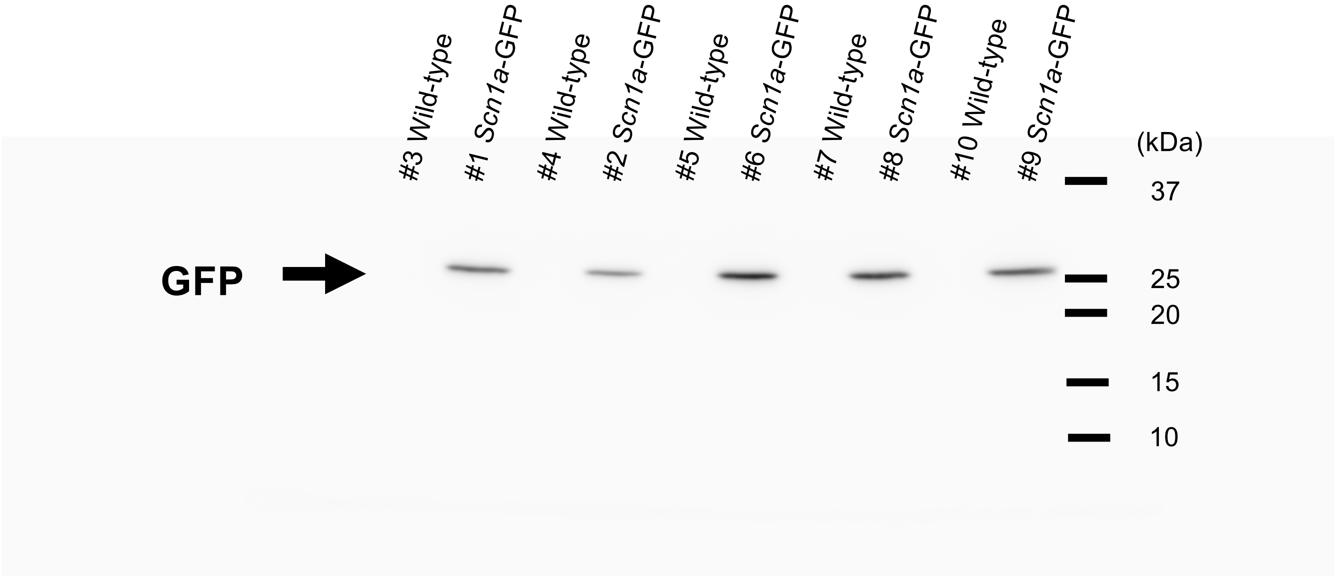

Figure 2-figure supplement 2-source data1-4

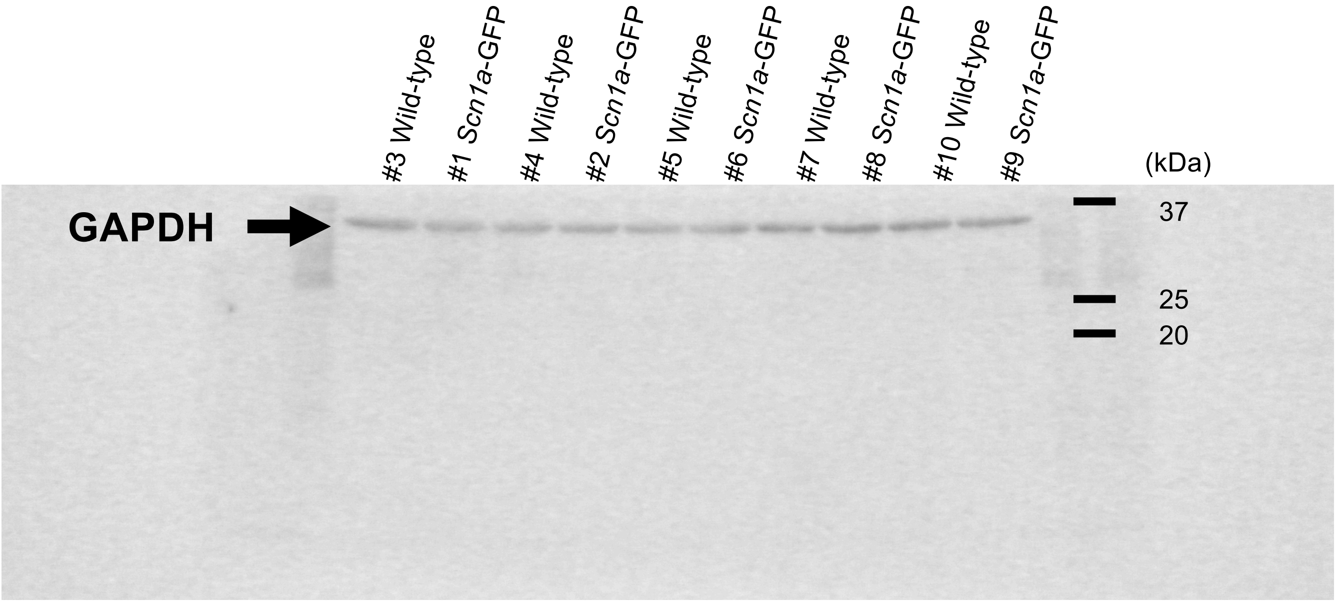

Supplement: Figure 2—figure supplement 2—source data 1. [file elife-87495-fig2-figsupp2-data1.zip › Figure2-figure_supplement_2-source data_1/Figure2-figure_supplement_2_source data1.pdf]
